# Effects of high doses of glucocorticoids on insulin-mediated vasodilation in the mesenteric artery of rats

**DOI:** 10.1371/journal.pone.0230514

**Published:** 2020-03-18

**Authors:** João Eliakim dos S. Araujo, Rodrigo Miguel-dos-Santos, Fabrício N. Macedo, Patrícia S. Cunha, Milene Tavares Fontes, Gilson Masahiro Murata, Sandra Lauton-Santos, Valter J. Santana-Filho, Ana Mara de O. Silva, Angelo Roberto Antoniolli, Rui Curi, Jullyana de S. S. Quintans, Rosana de S. S. Barreto, Marcio R. V. Santos, Lucindo J. Quintans-Junior, André S. Barreto

**Affiliations:** 1 Laboratory of Cardiovascular Pharmacology, Department of Physiology, Federal University of Sergipe, Sao Cristovao, Sergipe, Brazil; 2 Laboratory of Cardiovascular Biology and Oxidative Stress, Department of Physiology, Federal University of Sergipe, Sao Cristovao, Sergipe, Brazil; 3 Faculdade Estacio de Sergipe, Aracaju, Sergipe, Brazil; 4 Vascular Physiology Laboratory, Department of Physiology and Biophysics, Institute of Biomedical Sciences, University of São Paulo, São Paulo, Brazil; 5 Department of Physiology and Biophysics, Institute of Biomedical Sciences, University of São Paulo, São Paulo, Brazil; University of Southampton, UNITED KINGDOM

## Abstract

Several pathological conditions predict the use of glucocorticoids for the management of the inflammatory response; however, chronic or high dose glucocorticoid treatment is associated with hyperglycemia, hyperlipidemia, and insulin resistance and can be considered a risk factor for cardiovascular disease. Therefore, we investigated the mechanisms involved in the vascular responsiveness and inflammatory profile of mesenteric arteries of rats treated with high doses of glucocorticoids. Wistar rats were divided into a control (CO) group and a dexamethasone (DEX) group, that received dexamethasone for 7 days (2mg/kg/day, i.p.). Blood samples were used to assess the lipid profile and insulin tolerance. Vascular reactivity to Phenylephrine (Phe) and insulin, and O_2_^•-^production were evaluated. The intracellular insulin signaling pathway PI3K/AKT/eNOS and MAPK/ET-1 were investigated. Regarding the vascular inflammatory profile, TNF-α, IL-6, IL-1β and IL-18 were assessed. Dexamethasone-treated rats had decreased insulin tolerance test and endothelium-dependent vasodilation induced by insulin. eNOS inhibition caused vasoconstriction in the DEX group, which was abolished by the ET-A antagonist. Insulin-mediated relaxation in the DEX group was restored in the presence of the O_2_^.-^ scavenger TIRON. Nevertheless, in the DEX group there was an increase in Phe-induced vasoconstriction. In addition, the intracellular insulin signaling pathway PI3K/AKT/eNOS was impaired, decreasing NO bioavailability. Regarding superoxide anion generation, there was an increase in the DEX group, and all measured proinflammatory cytokines were also augmented in the DEX group. In addition, the DEX-group presented an increase in low-density lipoprotein cholesterol (LDL-c) and total cholesterol (TC) and reduced high-density lipoprotein cholesterol (HDL-c) levels. In summary, treatment with high doses of dexamethasone promoted changes in insulin-induced vasodilation, through the reduction of NO bioavailability and an increase in vasoconstriction via ET-1 associated with generation of O_2_^•-^ and proinflammatory cytokines.

## Introduction

Glucocorticoids (GC) have been widely used due to their antiallergic and anti-inflammatory properties; however, even a single dose can alter carbohydrate and lipid metabolism. In addition, chronic use may lead to various side effects such as changes in lipid, protein and carbohydrate metabolism, resulting in dyslipidemia, hyperglycemia, hyperlipidemia and insulin resistance [1,2]. This has been shown in clinical trials during mental stress and in patients with Cushing's syndrome [3]. These changes in glucose and insulin concentrations may be partially explained through effects on the insulin signaling pathway in both hepatic and extrahepatic cells [4,5], which can develop insulin resistance (IR). IR is considered a risk factor for cardiovascular diseases, such as myocardial infarction, atherosclerosis [6] and hypertension [7–9], as well as peripheral vascular disease, due to the damage caused to the vascular endothelium, increasing cardiovascular morbidity and mortality [6,10]. The insulin-signaling pathways regulate endothelial production of NO through binding to its receptor tyrosine kinase, resulting in the phosphorylation of the insulin receptor substrate (IRS-1), which then binds and activates phosphatidylinositol 3-kinase (PI3K), stimulating Akt activity. Akt directly phosphorylates eNOS of the Ser1177 residue, resulting in increased eNOS activity and subsequent NO production [11,12]. In addition to stimulating the production of NO through the PI-3K/Akt pathway, insulin also stimulates the production of the potent vasoconstrictor ET-1 through a separate mitogen-activated protein kinase (MAPK) pathway, in which it limits eNOS activity, impairing NO bioavailability [11–13]. Apart from its direct vasomotor activity, overproduction of ET-1 is associated with increased reactive oxygen species (ROS) production and inflammatory processes within the vascular wall, which are of importance in the atherosclerotic process, endothelial dysfunction and future cardiovascular events [14,15]. Some studies have demonstrated that glucocorticoids, besides promoting IR, can lead to compromised endothelial function in response to acetylcholine [16,17]. However, other studies using different doses of glucocorticoids and time-course of treatment have not fully confirmed this hypothesis [18–21]. Therefore, the effects of GC on vascular function are still not entirely elucidated, as the literature shows quite contradictory results regarding the vasodilator pathways affected and the mechanisms by which glucocorticoids impair vasodilation. The present study aimed to investigate the effects of high dose glucocorticoid treatment of rats on mechanisms of tone regulation and inflammatory profile in mesenteric arteries.

## Material and methods

### Animals

Adult male Wistar rats (300-350g) were obtained from the Central Animal Facility of the Federal University of Sergipe. Rats were kept in collective cages (5 animals/cage), in a temperature-controlled room (22 ± 2°C) with a 12 h light/12 h dark cycle, and received commercial rodent chow (Presence®) and filtered water ad libitum. The rats were randomized into two groups: control (CO, n = 20), and treated with dexamethasone (DEX, n = 20). Rats were submitted to a daily intraperitoneal injection of dexamethasone (DEX, 2.0 mg/kg/day) and performed alternately in different sides for 7 days. This dose has previously been prescribed in healthy humans [21]. The CO group was injected with 0.9% of NaCl using the same administration protocol as the DEX group. All procedures described in this study are in agreement with the guidelines of the Brazilian Society of Laboratory Animal Science and were approved by the Ethics Committee on Animal Research of the Federal University of Sergipe, Brazil (protocol number 75/2015).

### Measurement of metabolic parameters

Eight-hour fasting glucose level was measured on the eighth day after the beginning of the insulin resistance induction protocol. Blood was obtained by caudal puncture and a glucometer was used to determine the plasma glucose (Accu-Chek Advantage II, Roche, São Paulo, SP, Brazil).

After measuring fasting glucose, the animals were anesthetized with isoflurane and then euthanized by exsanguination. Blood samples were then collected and centrifuged at 5,000 g for 10 min at 4°C and stored at -80°C until they were analyzed. Blood samples were used to measure total cholesterol (TC), high-density lipoprotein cholesterol (HDL-c), and triglyceride (TG) concentrations by means of a commercial kit (Bioclin, Belo Horizonte, MG, Brazil). Levels of low-density lipoprotein cholesterol (LDL-c) were obtained by the Friedwald calculation [22].

On the 8th day insulin sensitivity was determined using the Insulin Tolerance Test (ITT). Rats were not fed for 6 h and were then injected with 0.75 U/kg body weight of human regular insulin (100 UI/ml). Blood samples were obtained via the tail vein at 0, 30, 60 and 120 min for subsequent measures of glucose [23].

### Vascular reactivity studies

Following animal sacrifice, the superior mesenteric artery was removed, and loose connective tissue and fat were carefully removed and the artery was sectioned into rings (1–2 mm). The rings were suspended from fine stainless-steel hooks, connected to a force transducer (Letica, Model TRI210; Barcelona, Spain) coupled to an amplifier-recorder (BD-01, AVS, SP, Brazil) with cotton threads in organ baths containing 10 mL of Tyrode's solution (composition in mM: NaCl 158.3, KCl 4.0, CaCl_2_ 2.0, NaHCO_3_ 10.0, glucose 5.6, MgCl_2_ 1.05 and NaH_2_PO_4_ 0.42). This solution was continually gassed with carbogen (95% O_2_ and 5% CO_2_) and maintained at 37°C under a resting isometric tension of 0.75 g for 60 min (stabilization period). During this time, the nutrient solution was changed every 15 min to prevent the interference of metabolites [24]. The functionality of the endothelium was assessed by the ability of acetylcholine (ACh, 1 μM) to induce more than 75% relaxation of phenylephrine (Phe, 1 μM)-induced pre-contraction. The vessel contractility was tested by exposure to Tyrode's solution with a high potassium concentration solution (KCl 80 mM, composition in mM: NaCl 82.3, KCl 80.0, CaCl_2_ 2.0, MgCl_2_ 1.05, NaHCO_3_ 10.0, NaH_2_PO_4_ 0.42, glucose 5.6), which was made equimolar by replacement of NaCl with KCl. The contraction induced by the high-K^+^ solution was similar between the groups: CO: 0.52 ± 0.07grams (n = 8) vs. DEX: 0.55 ± 0.09 grams (n = 9). Changes in vascular reactivity were then assessed by obtaining concentration-response curves for insulin (10^−13^–10^−6^ M). These curves were obtained after incubation for 30 min with the following inhibitors: L-NAME was used to evaluate the role of NO (inhibitor of nitric oxide synthase; 100 μM); L-NAME + BQ123, to evaluate the role of endothelin-1 (a selective ETA receptor antagonist; 1 μM); LY294002, to evaluate the role of the PI3K pathway (inhibitor of PI3K; 50 μM) and TIRON, to evaluate the role of O_2_^•-^ (O_2_^•-^ scavenger: 20 μM). In addition, concentration-response curves to Phe were performed (10^−9^–3x10^−5^ M).

### Western blot analysis

Western blot was performed as described previously [25]. Mesenteric artery was homogenized in ice-cold lysis buffer containing (in mM): 150 NaCl, 50 Tris-HCl, 5 EDTA.2 Na and 1 MgCl2, pH 8.0; 1% Triton X-100, 1% NP-40, 1% sodium deoxycholate, 0.1% sodium dodecyl sulfate enriched with a protease inhibitor cocktail (Sigma FAST, Sigma, St. Louis, MO). Homogenates were cleared by centrifugation at 13,000 × g for 15 min at 4°C and protein content was quantified by Lowry assay. Samples were denatured in Laemmli buffer and an equal amount of protein (30 μg/lane) was separated on 7.5% SDS-polyacrylamide gel on electrophoresis and then transferred onto a nitrocellulose membrane (2 h at 120 V, Merck-Millipore, Billerica, MA). Membranes were blocked for 2 h in Tris-buffered saline-Tween 20 containing 5% non-fat dry milk at room temperature before incubation with rabbit polyclonal anti- PI3K, anti-Akt, anti-eNOS, and goat polyclonal anti-β-actin antibodies (1:1000; Santa Cruz Biotechnology, Santa Cruz, CA) overnight at 4°C. After washing, membranes were incubated for 2 h at room temperature with anti-rabbit or anti-goat IgG-HRP antibody (1:10,000, Sigma-Aldrich, St. Louis, Missouri, USA) and immunodetection was performed using enhanced chemiluminescence (Luminata strong^™^-Western HRP substrate, Merck-Millipore, Billerica, MA). Digitalized images were analyzed by densitometry using ImageJ software (NIH).

### Measurement of NO production

NO production in the mesenteric artery ring was determined using a fluorescent cell permeable dye for NO, 4,-amino-5 methylamino-2′, 7′-diaminofluorescein diacetate (DAF-FM, Molecular Probes), as previously described [26]. In order to detect NO, the fresh mesenteric artery was loaded at 37°C with 10 μmol/L of the probe for 20 min, then some rings were stimulated with 10^−7^ M of regular human insulin, with or without L-NAME for 20 min, and washed for 40 min with Tyrode's solution. Mesenteric segments were snap-frozen and cut into 20 μm thick slices. Images were recorded using fluorescence microscopy (IX2-ICB, Olympus®, USA). Analyses of the images were performed using Image J software (NIH).

### Measurement of O_2_^•-^ production

Measurement of superoxide anion (O_2_^•-^) production in the mesenteric artery rings was determined using a fluorescent cell-permeable dye for O_2_^•-^, dihydroethidium (DHE, Molecular Probes), as previously described [27]. In order to detect O_2_^•-^, the fresh mesenteric artery was loaded at 37°C with 10 μmol/L of the probe for 20 min, then some rings were stimulated with 10^−7^ M of regular human insulin for 20 min, and washed for 40 min with Tyrode's solution. Mesenteric segments were snap frozen and cut into 20 μm thick slices. Images were recorded using a fluorescence microscope (IX2-ICB, Olympus®, USA). Analyses of images were performed in Image J software (NIH).

### Measurement of pro-inflammatory cytokines (IL-6, IL-1β, TNF-α and IL-18)

Inflammatory cytokine expression was evaluated by RT-PCR. Total RNA was extracted using Trizol reagent as described by the manufacturer (Invitrogen). The samples were lysed using 1 ml Trizol reagent (Invitrogen) and after 15 min of incubation at room temperature, 200 μl chloroform was added to the tubes, mixed and centrifuged at 12,000g. The aqueous phase was transferred to another tube, and the RNA was pelleted with cold isopropanol by centrifugation (12,000g) and dried in air. RNA pellets were eluted in RNase-free water and RNA integrity was assessed by 260/268 nm ratio and on a TAE 1% agarose gel electrophoresis with blue-green stain (LCG biotechnology).

cDNA was synthesized from 2 μg total RNA after DNAse treatment using a High Capacity Reverse Transcription kit (Thermo Fischer Scientific), as described by the manufacturer. RT- PCR was performed in a Rotor-Gene 3000 (Corbett Research, Mortlake, Australia) machine, with the Platinum® SYBR® Green qPCR SuperMix UDG kit (Thermo Fischer Scientific) (Table 1). Quantification was performed by the 2−ΔΔCT method, using 18S rRNA as the housekeeping gene. The PCR cycling conditions for all reactions were 95°C for 30 s, 60°C for 30 s, and 72°C for 30 s.

**Table 1:** Primer sequences (PCR-RT SYBR green).

Gene	Primer sequences	
	Sense	Antisense
TNF-α	TCTTCTCATTCCTGCTTGTGGC	CACTTGGTGGTTTGCTACGACG
IL-1β	AAATGCCTCGTGCTGTCTGA	AGGCCACAGGGATTTTGTCG
IL-6	CCTTCTTGGGACTGATGTTGTTGAC	GGGTGGTATCCTCTGTGAAGTCTCC
IL-18 Leptin	AACCGCAGTAATACGGAGCAT GTGGCTATCCACAAAGTCCAGG	CGTTGGCTGTTCGGTCGATA CGCAGGTTCTCCAGGTCATG

### Statistical analysis

All data are expressed as mean ± S.E.M. Significant differences between groups were determined using Two-way ANOVA followed by Bonferroni’s post hoc test to compare the concentration-response curves obtained in the mesenteric rings (n = 6 animals in each group) and the results of the insulin sensitivity test (n = 6 animals in each group). One-way ANOVA followed by Bonferroni’s post hoc test was used to compare the NO production (n = 4 animals in each group) and superoxide production (n = 4 animals in each group). Student’s unpaired *t*-test was used to compare metabolic parameters (n = 6 animals in each group), PI3K, Akt and eNOS expression (n = 4 animals in each group) and mRNA expression of pro-inflammatory cytokines (n = 3 animals in each group). All statistical comparisons were made using GraphPad Prism 5.1 (GraphPad Software Inc., San Diego, CA, USA) and values of p<0.05 were considered to be statistically significant.

## Results

### Body weight and metabolic parameters

The results of body weight and metabolic parameters are shown in Table 2. The body weight of the animals was similar in all groups at the beginning of the study. Final body weight was significantly reduced in the DEX group compared to baseline (p<0.05) and to the CO group (p<0.001). Moreover, the DEX group had increased glycaemia (p<0.001), TC (p<0.01), LDL (p<0.01) and decreased HDL (p<0.01) compared with the CO group. No significant difference was observed in triglyceride levels.

**Table 2:** Bodyweight in the first and eighth week, fasting glucose, TG, TC, LDL and HDL.

Group		CO	DEX
Body weight (g)	Initial	335.83 ± 4.6	335.33 ± 1.9
	Final	337.33 ± 3.8	271.50 ± 5.0***^#^
Fasting glucose (mg/dL)	Final	96.6 ± 1.2	127.8 ± 4.2***
TGs (mg/dL)	Final	96.3 ± 5.6	109.1 ± 4.23
TC (mg/dL)	Final	166.8 ± 6.0	237.88 ± 12.7**
LDL-c (mg/dL)	Final	100.3 ± 9.0	176.1 ± 13.3**
HDL-c (mg/dL)	Final	56.09 ± 4.5	37.5 ± 2.8**

CO, Control group; Insulin resistance (DEX) group. All of the values are the mean ± S.E.M., (n = 6). For data analysis, one-way ANOVA was used followed by Bonferroni post-test for the bodyweight and Student’s unpaired t-test was used for the fasting glucose, triglycerides (TG), total cholesterol (TC), low-density lipoprotein cholesterol (LDL-c) and high-density lipoprotein cholesterol (HDL-c).

**p < 0.01 and

***p < 0.001 *vs*. CO

^#^p < 0.001, DEX initial *vs*. DEX final.

**Fig 1:**
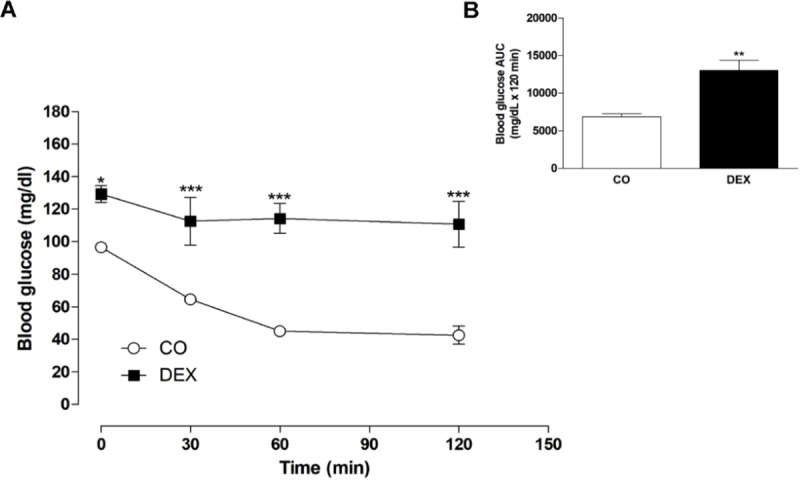
(A) Insulin tolerance test (ITT) and (B) area under the curve (AUC) data for blood glucose. Values are expressed as mean ± S.E.M. For data analysis, one-way ANOVA followed by Bonferroni post-test was used for the ITT, and Student’s unpaired t-test was used for the AUC. *p< 0.05, **p< 0.01, ***p< 0.001, CO vs DEX.

Concerning insulin sensitivity evaluated by ITT, there was elevated blood glucose at all time points (0, 30, 60 and 120 min, p<0.001) in the DEX group (Fig 1A). This was confirmed through AUC assessment (p<0.01) in which there was an increase of 52% in the DEX group (Fig 1B).

### Endothelium and non-endothelium dependent mechanisms of vasodilation and vasoconstriction

**Fig 2:**
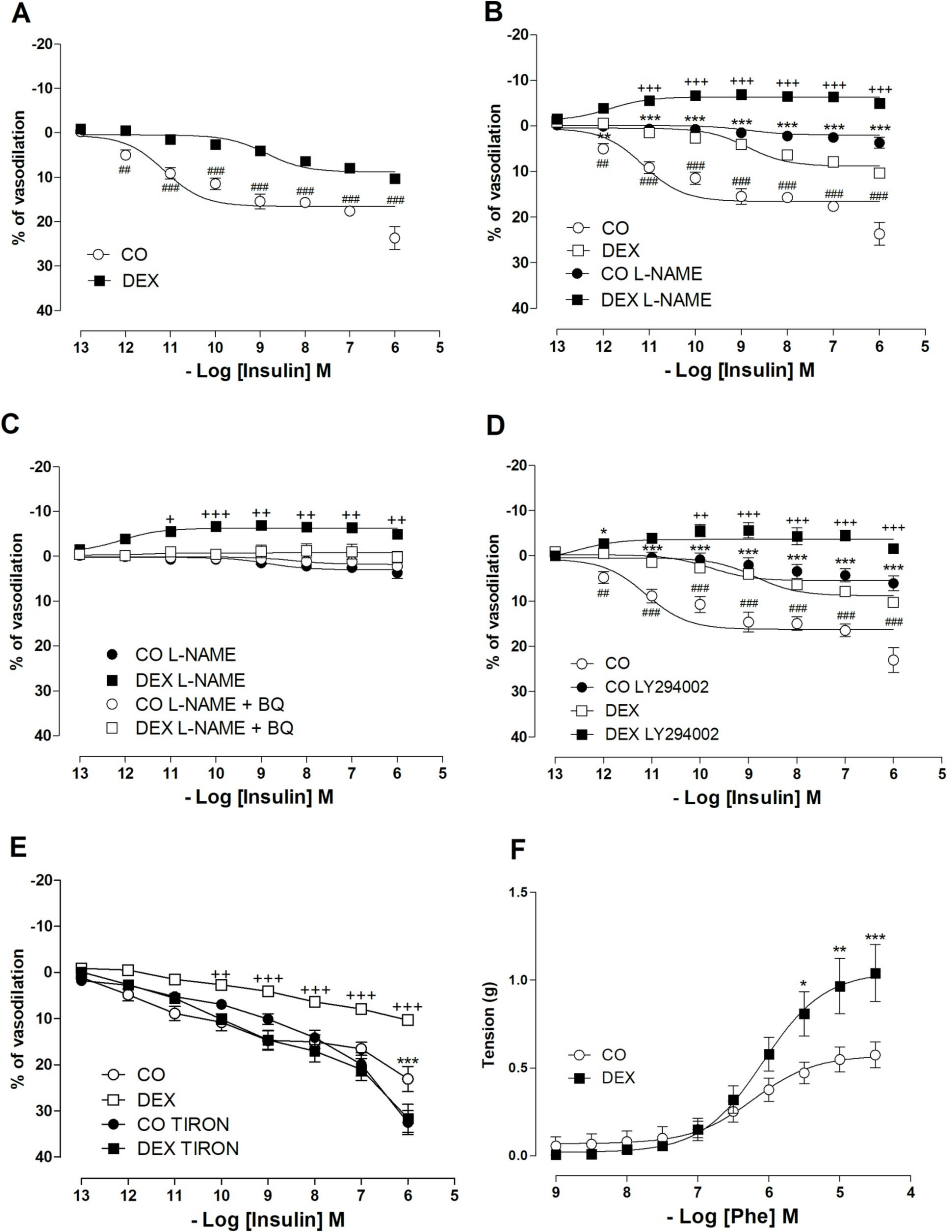
Endothelium-dependent relaxation in response to insulin (INS) in control and insulin-resistant rats. Cumulative concentration-response curves to insulin in intact segments obtained from the superior mesenteric artery of Wistar rats and pre-contracted with phenylephrine (1 μM) in the control (CO) and dexamethasone-treated (DEX) groups (A). These graphs are reproduced in panels B and D to permit comparison with those obtained in the presence of L-NAME (100 μM) (B) or LY294002 (50 μM) (D). Responses obtained in the presence of L-NAME and in the absence or presence of BQ123 (50 μM) (C). Responses obtained in the absence and presence of TIRON (20 μM) (E) and cumulative concentration-response curve for Phe (10^−9^–3x10^−5^ M) (F). The results are expressed as the mean ± SEM for n = 6 animals in each group. For data analysis, two-way ANOVA followed by Bonferroni post-test were used. A: ^##^p< 0.01, ^###^p< 0.001, CO vs. DEX; B: ***p< 0.001, CO vs. CO L-NAME and ^+++^p<0.001, DEX vs DEX L-NAME; C: ^+^p< 0.05, ^++^p< 0.01, ^+++^p<0.001, DEX L-NAME vs. DEX L-NAME + BQ123; D: ***p< 0.001, CO vs. CO L294002, ^++^p< 0.01, ^+++^p<0.001, CO L294002 vs. DEX L294002; E: ***p< 0.001, CO vs. CO TIRON; ^++^p< 0.01, ^+++^p<0.001, DEX vs. DEX TIRON; F: *p< 0.05, CO vs. DEX; **p< 0.01, CO vs. DEX.; ***p< 0.001, CO vs. DEX.

Insulin-induced vasodilatation was reduced in the DEX group (R_max_ = 23.6 ± 2.5 vs 10.3 ± 0.7%; p < 0.001) (Fig 2A). Maximal Phe-induced vasoconstriction was significantly increased in the DEX group in relation to CO (R_max_ = 1.04 ± 0.16 vs 0.57 ± 0.07g, p<0.05) as shown in Fig 2F.

### Changes in the signaling pathway of insulin-induced vasodilation

To evaluate NO participation in the vasodilation induced by insulin, a non-selective inhibitor of NOS (L-NAME) was used. After incubation with L-NAME a reduction of relaxation was observed in the CO group (R_max_ = 23.6 ± 2.5% to 3.7 ± 1.1%, p < 0.001; Fig 2B), whereas in the DEX group the vasodilation was abolished and replaced with small amplitude contraction (Before: R_max_ = 10.3 ± 0.7% vs after: R_max =_ -4.9 ± 0.7%, p < 0.001; Fig 2B).

In order to understand the participation of ET-1 in this response, a concentration-response curve in the presence of L-NAME + BQ123 (an antagonist of ETA receptors) was constructed. The CO group showed no further change in R_max_ (3.7 ± 1.1% to 2.2 ± 1.1%, p>0.05; Fig 2C). However, in the DEX group the contraction in the presence of L-NAME alone was abolished by co-incubation with BQ123 was abolished (R_max_ = -4.9 ± 0.7% to 0.1 ± 1.3%, p < 0.05; Fig 2C). Furthermore, to evaluate PI3K participation in the vasodilatation induced by insulin, an inhibitor of PI3K (LY294002) was used. After incubation with LY294002 a reduction of relaxation was observed in the CO group (Rmax = 23.6 ± 2.5% to 6.1 ± 1.6%, p < 0.001; Fig 2D), whereas in the DEX group the vasodilation was totally abolished, showing a slight contractile effect (Before: Rmax = 10.3 ± 0.7% vs after: Rmax = -1.6 ± 0.8%, p < 0.001; Fig 2D).

After this set of experiments, we performed a concentration-response curve in the presence of TIRON, a scavenger of superoxide anions. TIRON increased the vasorelaxation in the CO group (R_max_ = 23.6 ± 2.5% to 32.4 ± 2.6%, p < 0.05) and restored the vasodilation to insulin in the DEX group (R_max_ = 10.3 ± 0.7% to 31.5 ± 3.0%, p < 0.001; Fig 2E).

### Molecular alterations in the PI3K/Akt/eNOS pathway

**Fig 3:**
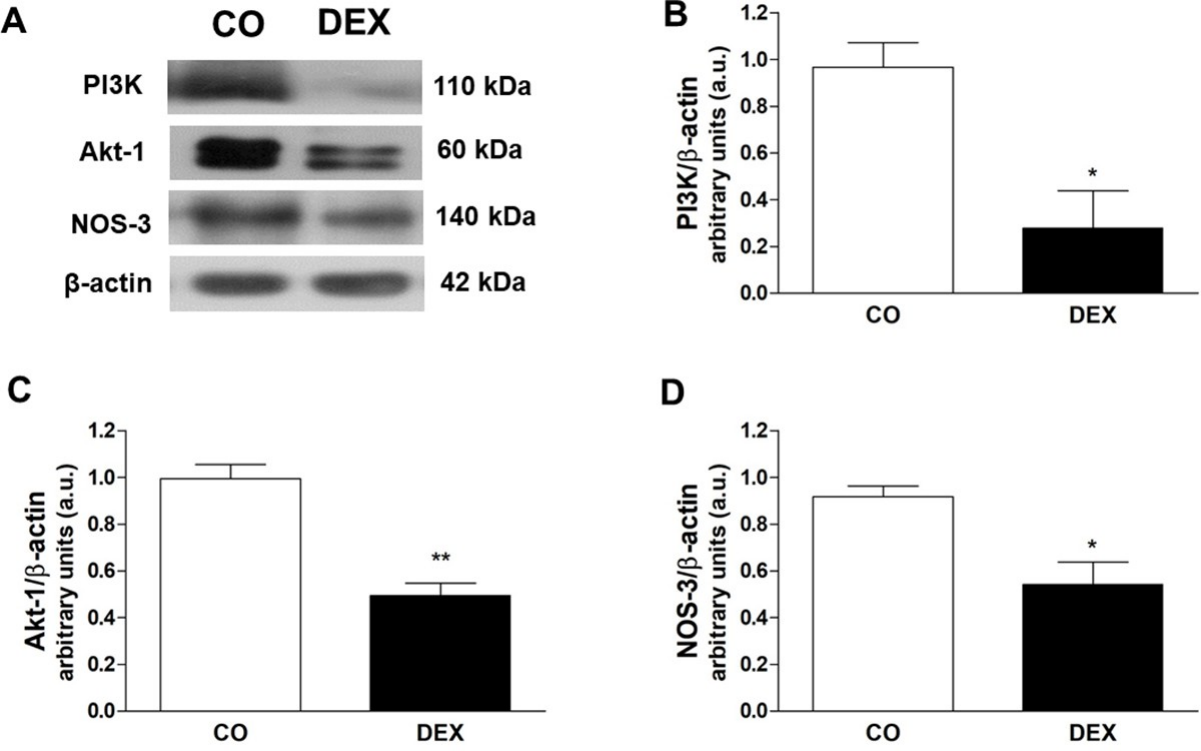
Effects of insulin resistance on PI3K, Akt and eNOS expression. (A), representative images of western blot and (B-D), quantitative analysis of total PI3K, Akt and eNOS protein levels in mesenteric artery of control (CO) and dexamethasone-treated (DEX) groups. The results are expressed as the mean ± SEM for 4 different samples from different animals in each group. For data analysis, Student’s unpaired t-test was used. *p<0.05, **p<0.01, CO vs DEX.

Based on the results of the vascular reactivity experiments, we evaluated whether treatment with DEX alters the protein expression of PI3K, Akt and eNOS in the mesenteric artery. In the DEX group, there were significant reductions in protein expression of PI3K (0.27 ± 0.16 vs 0.96 ± 0.1 a.u., p < 0.05), Akt (0.49 ± 0.05 vs 0.99 ± 0.06, p < 0.01) and eNOS (0.54 ± 0.09 vs 0.91 ± 0.04 a.u., p < 0.05), compared to the CO group (Fig 3).

### NO bioavailability under basal and insulin-stimulated conditions in the absence or presence of L-NAME

**Fig 4:**
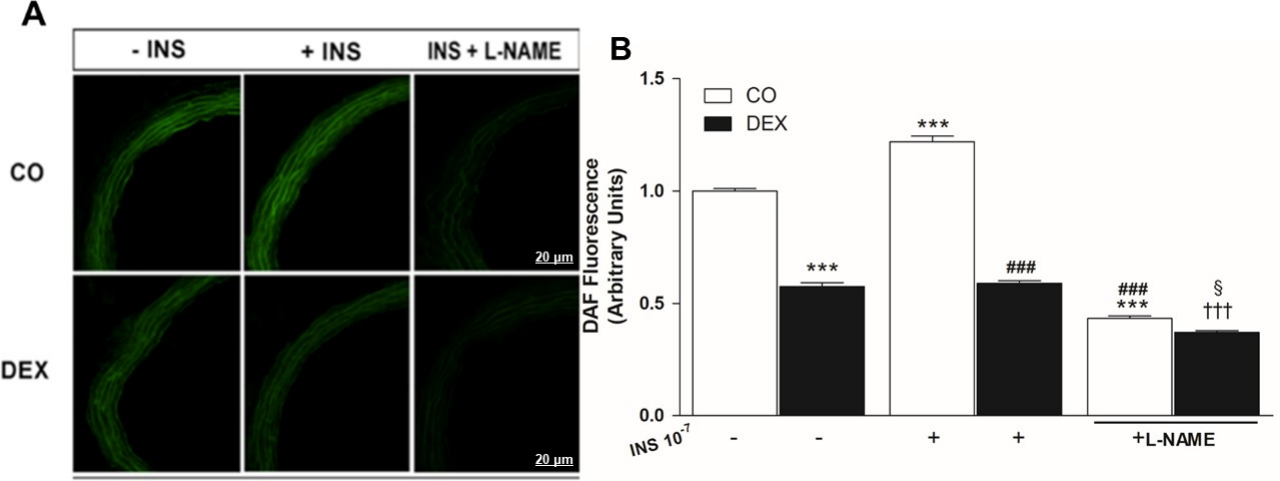
Effects of insulin on nitric oxide (NO) production in the superior mesenteric artery. (A) Representative images of DAF fluorescence, a NO indicator and (B), quantification of DAF fluorescence in a superior mesenteric artery of control (CO) and dexamethasone treated (DEX) rats in the presence and absence of insulin or presence and absence of L-NAME. 20 to 30 slices of each ring of the superior mesenteric artery were analyzed (n = 4 animals). Scale bar = 20 μm. Values are expressed as mean ± S.E.M. For data analysis, one-way ANOVA followed by Bonferroni post-test was used. ***p<0.001, vs CO under basal condition (-); ^###^p<0.001 vs CO under INS stimulation (+); ^†††^p<0.001 vs DEX under INS stimulation (+);^§^p<0.05, vs CO under INS stimulation + L-NAME (+).

Considering the in vitro findings and lower PI3K/Akt/eNOS pathway expression, we assessed whether DEX affects NO synthesis in the mesenteric artery. Interestingly, NO bioavailability under basal conditions was higher in the mesenteric arteries from the CO group compared to the DEX group (1.0 ± 0.01 to 0.57 ± 0.01 a.u., ***p<0.001; Fig 4).

After insulin stimulation the CO group showed augmented NO bioavailability (1.21 ± 0.02 a.u., ***p<0.001) when compared to basal conditions, however, in the DEX group no changes were observed (0.58 ± 0.01 a.u., p>0.05). In addition, insulin stimulation elicited a smaller increase in fluorescence in the DEX compared with the CO group (p < 0.001). The preincubation with L-NAME reduced NO bioavailability in both groups (CO: from 1.21 ± 0.02 to 0.43 ± 0.01 a.u. and DEX: from 0.58 ±0.01 to 0.37 ± 0.01 a.u., ^###, †††^p<0.001). However, the DEX group showed greater inhibition of NO (^§^p < 0.05; Fig 4).

### Superoxide production under basal and insulin-stimulated conditions

**Fig 5:**
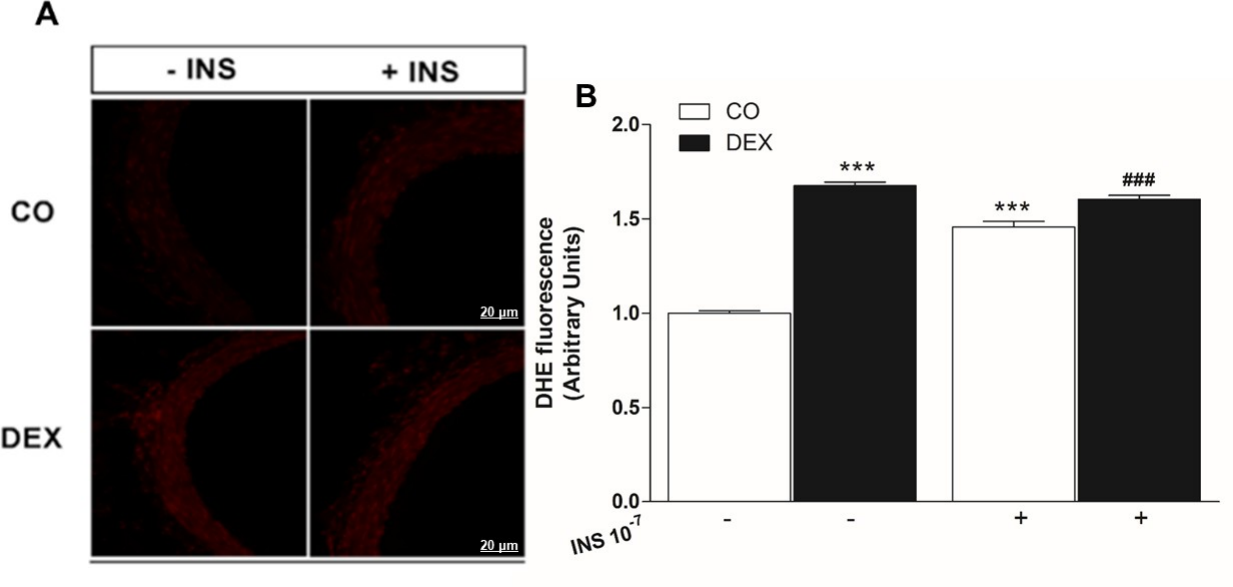
Effects of insulin on superoxide anion (O2•-) production in the superior mesenteric artery. (A) Representative images of DHE fluorescence, a ROS indicator and (B), quantification of DHE fluorescence in a superior mesenteric artery of control (CO) and dexamethasone treated (DEX) rats in the absence and presence of insulin. n = 10 superior mesenteric rings analyzed from 4 animals. Scale bar = 20 μm. Values are expressed as mean ± S.E.M. For data analysis, one-way ANOVA followed by Bonferroni post-test was used. ***p<0.001, vs CO under basal condition (-); ###p<0.001 vs CO under INS stimulation (+).

At baseline, DHE fluorescence analysis revealed an increase in superoxide anion production in the DEX group compared to CO group (1.0 ± 0.01 to 1.67 ± 0.01 a.u., p<0.001). After insulin stimulation, there was enhanced endothelial O_2_^•-^ generation in the CO group (1.45 ± 0.02 a.u., p<0.001), but not in the DEX group (1.60 ± 0.02 a.u., p>0.05) when compared to their respective basal conditions (Fig 5). Moreover, the DEX group showed higher superoxide generation after stimulation with insulin when compared to CO group (p<0.001).

### mRNA expression of pro-inflammatory cytokines (IL-6, IL-1β, TNF-α and IL-18)

**Fig 6:**
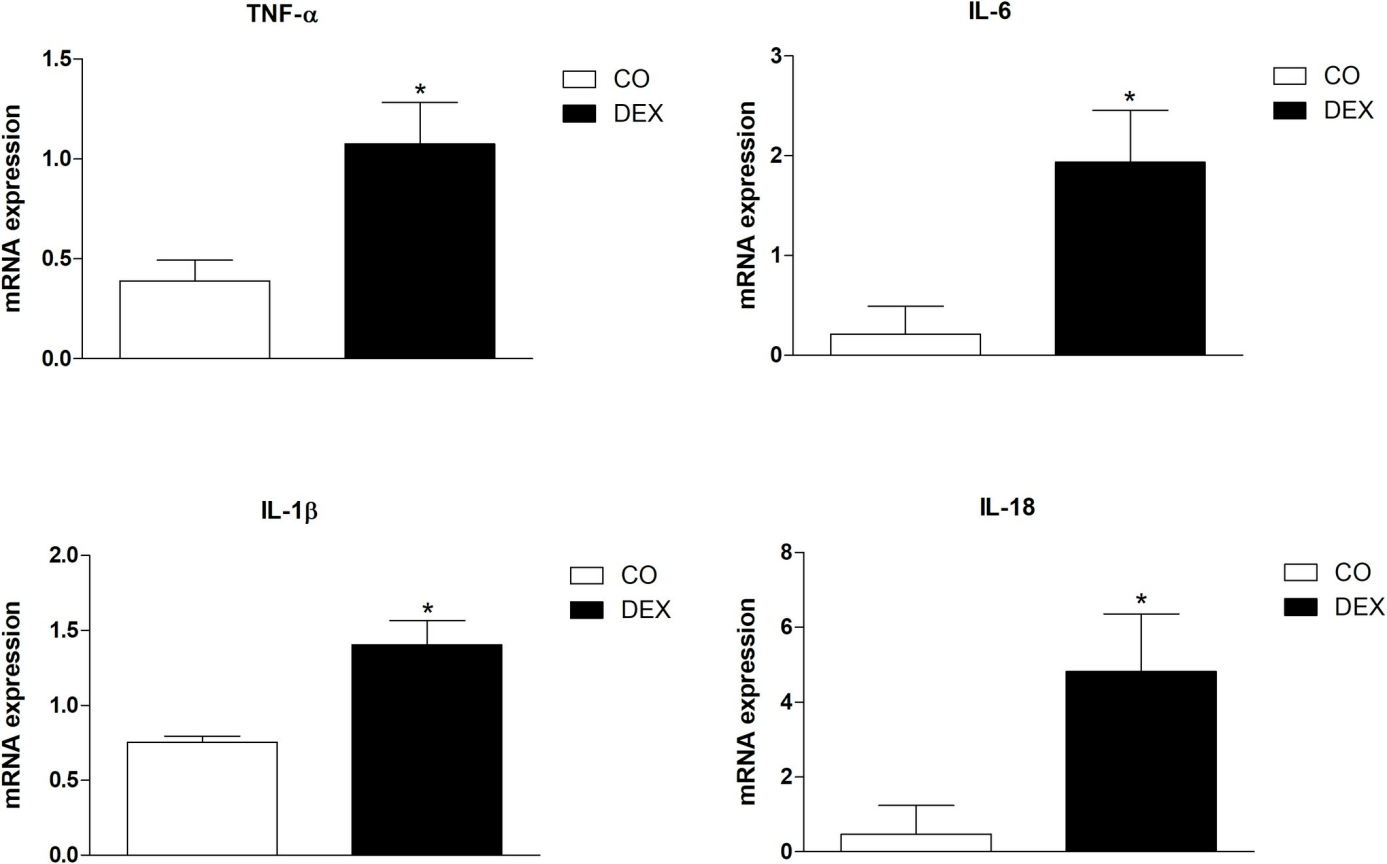
Effects of insulin resistance on pro-inflammatory cytokine gene expression in mesenteric artery of control (CO) and dexamethasone treated (DEX) groups. The results are expressed as the mean ± SEM, n = 3. For data analysis, Student’s unpaired t-test was used. *p<0.05, **p<0.01 CO vs DEX.

Fig 6 shows the expression of pro-inflammatory cytokines in mesenteric artery. There was an increase in the DEX group in mRNA expression of IL-6, IL-1β, TNF-α and IL-18, compared to the CO group.

## Discussion

In the present study, we evaluated the effect of glucocorticoid use on vascular function through the insulin-signaling pathway. The main results of one week of administration of glucocorticoids were: (1) Metabolic dysfunction described by augmentation of fasting glycaemia, TC, LDL-c and reduction in HDL-c plus insulin resistance (2) Impairment of insulin-mediated vasodilation; (3) Involvement of PI3K/Akt/eNOS vasodilatory pathway in augmentation of ET-1-induced vasoconstriction; (4) Reduced endothelial NO bioavailability and increased O_2_^•-^ and (5) Increased gene expression of pro-inflammatory cytokines IL-6, IL-1β, TNF-α and IL-18.

In this study TC and LDL increased and HDL decreased, showing modifications in lipid metabolism. High-dose GC increased the triglyceride breakdown and release of glycerol and free fatty acids (FFA) through lipolysis. Glycerol contributes to increased hepatic gluconeogenesis, increasing glycaemia. Moreover, FFA becomes the primary substrate in the process of energy formation, and therefore glucose becomes a secondary energy substrate contributing to increased glycemia [28]. Furthermore, GC impairs the plasma lipid profile [29,30] through the increment of both TG and total LDL-c, and reduction of HDL-c concentrations. Despite this, other studies in animals using higher doses of dexamethasone for seven days (5 mg/kg/day) or in humans treated with 2 mg/kg/day of prednisolone orally for 4 weeks, have not shown differences in the levels of HDL, LDL and TC [21,31]. In the present study, although the dose or days of treatment used were not similar to the studies cited, the treatment route may justify this difference between the results.

These changes in the lipid profile caused by GCs can lead to increases in glycaemia, in addition to blunting intracellular insulin signal transduction [5]. In the present study there was increased glycaemia and reduced insulin sensitivity. IR, besides contributing to hyperglycemia, can also decrease the insulin-induced vasodilation and, potentiate its alternative vasoconstriction pathway. Taken together, the reduction of blood flow and glucose uptake by the blood vessels may generate endothelial dysfunction [11].

ACh-mediated endothelium-dependent relaxation is a prominent pathway in a variety of vascular beds. This pathway is characterized by increased release of [Ca^2+^]i binding to calmodulin, which activates eNOS and therefore elevates NO production. However, neither acute [19,20] nor chronic [21] glucocorticoid use appears to cause dysfunction of ACh-mediated endothelium-dependent vasodilation. Another endothelial pathway for vasodilation is insulin-mediated. Insulin participates directly in the maintenance of homeostasis and vascular tone, which can represent up to 25% of maximal vasodilation [13,24,26]. In our study, insulin-mediated vasodilatation was reduced in the DEX group. To our knowledge, this is the first study to observe changes in insulin-mediated vascular responsiveness after treatment with GCs. This pathway plays a key role in the maintenance of endothelial function, through stimulation of endothelial cells to produce NO via PI3K/Akt/eNOS. [13,24,26].

Thus, we evaluated the insulin-induced vascular effect in the presence of L-NAME and found that the DEX group showed vasoconstriction, the same effect was also found in the presence of LY294002. Studies have shown that treatment with GCs leads to a reduction of the tyrosine-phosphorylated insulin receptor and total IRS-1 proteins, decreasing activity of the PI3K and Akt in skeletal muscle [32]. Moreover, GCs inhibit IRS-1/PI3K/Akt signaling in renal epithelial cells [33], reducing the insulin response. To corroborate this hypothesis, we evaluated the total protein expression of PI3K and Akt. We observed a reduction in protein expression of both PI3K and Akt, and this was associated with impairment of NO release and vasodilation.

NO plays an important role in the control of vascular tone by acting as the main inducer of relaxation in vascular beds [12]. Regarding the NO participation on insulin-mediated vasodilation, we observed not only attenuation, but reversal of the concentration–response curve after eNOS inhibition in the DEX group. The attenuation of the insulin-mediated vasodilation in our study is due to reduction of NO bioavailability, mediated by a decrease in eNOS expression. Studies have demonstrated that GC treatment significantly down‐regulates eNOS expression [16,34]. These changes in eNOS expression following GC treatment may have been caused by uncoupling of eNOS, through inhibiting essential cofactors, becoming inactive and reducing NO production [35]. On the other hand, the reversal of the concentration response curve could be due to activation of vasoconstrictor mechanisms.

The literature shows that insulin pathway disturbance can induce vasoconstriction by endothelium-dependent mechanisms through activation of the MAPK/ET-1 pathway [36,37]. To evaluate this hypothesis, we used BQ123 + L-NAME simultaneously. Under these conditions, insulin-induced vasoconstriction was inhibited, suggesting that this effect is due to a predominance of MAPK/ET-1 in relation to the PI3K/eNOS pathway, which can lead to a reduction in NO bioavailability and increased ET-1 activation. Despite studies demonstrating enhancement of ET-1 concentration in the presence of GCs, it is likely that the ET-1 pathway is also upregulated [38,39]. Our study observed involvement of the ET-1 pathway; changes in insulin-mediated vascular responsiveness after treatment with GCs suggested involvement of ET-A receptor activation. Furthermore, we found an increase in contraction to phenylephrine. Similarly, other studies have also demonstrated that GCs enhanced Phe-induced vasoconstriction [40,41]. On the other hand, there is evidence that phenylephrine-induced vasoconstriction is attenuated in the presence of insulin in individuals using glucocorticoids [42]. Although these mechanisms have not been elucidated, it is possible that metabolic factors other than insulin dysfunction may be involved in increased vasoconstriction, such as dyslipidemia, ROS and pro-inflammatory cytokines [43].

GC-induced vasoconstriction may have occurred by an increase in the release of and sensitivity to [Ca^2+^]_i_, a determinant of smooth muscle contraction [40]. This increased contraction to Phe may have been caused by a reduction in PI3K/Akt/eNOS signaling, and is associated with impaired NO-dependent vasorelaxation, as can be seen in this study. In addition, the literature has reported that GCs increase signaling via the ET-1 pathway which can promote elevated vascular ROS production, especially O_2_^•-^. Thus, we performed experiments in the presence of TIRON (O_2_^•-^ scavenger) to evaluate the participation of ROS in the reduction of endothelium-dependent vasodilation. In this experiment, increased insulin-mediated vasodilation was observed in the DEX group, showing the importance of O_2_^•-^ in attenuation of vasodilation in this model. Vascular endothelial cells are capable of producing reactive oxygen species (ROS) such as O_2_^•-^, hydroxyl radicals and hydrogen peroxide. Intracellular overproduction of O_2_^•-^ can cause both eNOS uncoupling and react with NO and produce ONOO^-^, resulting in reduction in NO bioavailability [44].

Moreover, ROS production can induce an increase in pro-inflammatory markers, such as IL-1 β, IL-6 and TNF-⍺, reducing the action of insulin on target tissues [45–47]. In the present study, gene expression of IL-1β, IL-6, IL-18 and TNF-⍺ in mesenteric arteries was increased in the DEX group. Glucocorticoids are potent anti-inflammatory and immunosuppressive agents, prescribed widely for the treatment of both chronic inflammation and autoimmunity. In addition, studies have reported that GCs acting on endothelial cells tend to reduce expression of these cytokines [48] and inhibit atherosclerotic progression [49]. Nonetheless, excessive GC use increases pro-inflammatory gene expression (IL-6, IL-1β, TNF-α) [50,51], which can impair insulin sensitivity by increasing serine/threonine phosphorylation of the insulin receptor, which reduces insulin-induced tyrosine phosphorylation of IRS-1 and suppresses PI3K, resulting in decreased Akt phosphorylation and activation [52]. These changes lead to an imbalance between the PI3K/Akt/eNOS and MAPK/ET-1 pathways [52]. The raised gene expression of IL-1β, IL-6, IL-18 and TNF-⍺, caused by GC contributed to reduced insulin sensitivity, along with decreased NO production and increased insulin-mediated ET‐1 pathway signaling.

## Conclusion

In the present study, it was demonstrated that exposure to high doses of glucocorticoids promoted changes to metabolic parameters. In addition, there was impairment of the vasodilator PI3K/Akt/eNOS pathway and increased signaling through the vasoconstrictor MAPK/ET-1 pathway, and that was due, in part, to the reduction in NO bioavailability and increased O_2_^•-^ production, together with cytokine elevation. These changes to vascular endothelium in the presence of high doses of glucocorticoids can be an indicator for, or critical risk factor associated with, cardiovascular diseases.

## Supporting information

S1 Raw image (TIF)

S2 Raw image (TIF)
