# Supplementary figures and images for: Effects of high doses of glucocorticoids on insulin-mediated vasodilation in the mesenteric artery of rats
Source: PLoS One. 2020 Mar 18;15(3):e0230514. doi: 10.1371/journal.pone.0230514 (PMC7080254; doi:10.1371/journal.pone.0230514)

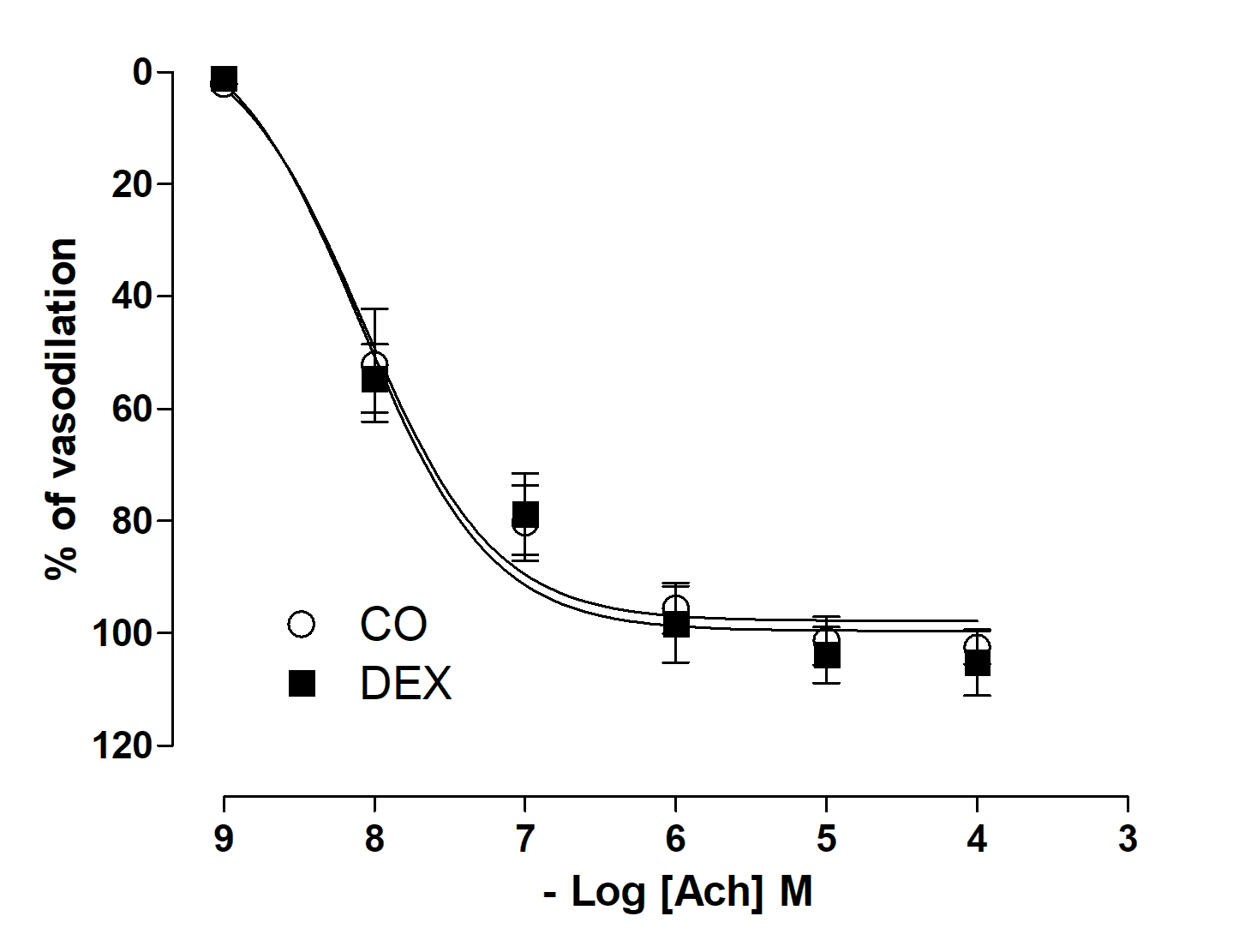

Supplement: S1 Raw image — (TIF) [file pone.0230514.s001.tif]

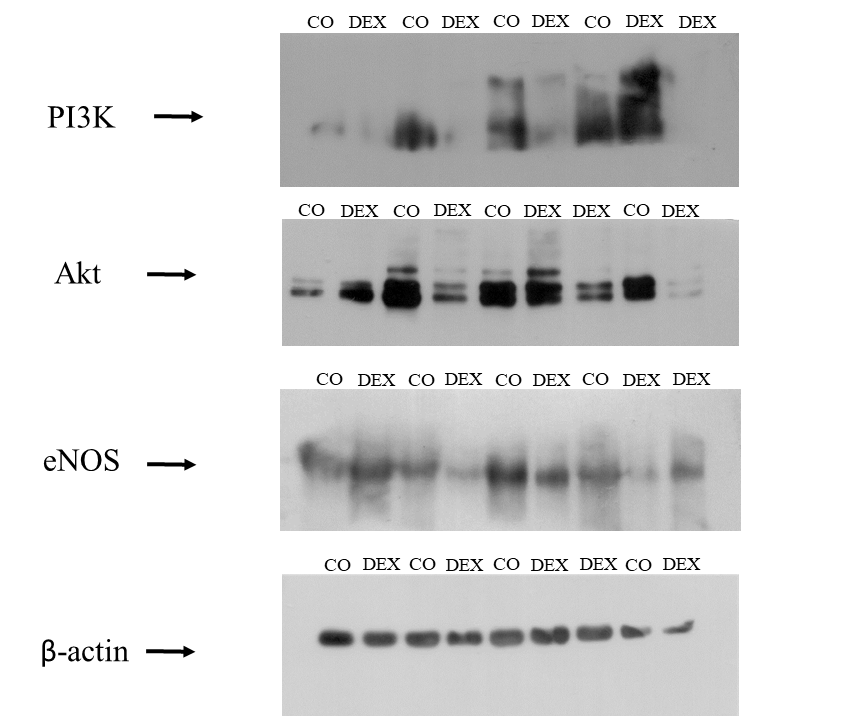

Supplement: S2 Raw image — (TIF) [file pone.0230514.s002.tif]
